# Holographic detection of nanoparticles using acoustically actuated nanolenses

**DOI:** 10.1038/s41467-019-13802-1

**Published:** 2020-01-16

**Authors:** Aniruddha Ray, Muhammad Arslan Khalid, Andriejus Demčenko, Mustafa Daloglu, Derek Tseng, Julien Reboud, Jonathan M. Cooper, Aydogan Ozcan

**Affiliations:** 1 Electrical and Computer Engineering Department, University of California, Los Angeles, CA 90095 USA; 2 Bioengineering Department, University of California, Los Angeles, CA 90095 USA; 3 California Nano Systems Institute (CNSI), University of California, Los Angeles, CA 90095 USA; 4 David Geffen School of Medicine, University of California, Los Angeles, CA 90095 USA; 5 Department of Physics and Astronomy, University of Toledo, Toledo, OH 43606 USA; 6 Division of Biomedical Engineering, James Watt School of Engineering, University of Glasgow, Glasgow, G12 8LT UK

**Keywords:** Biomedical engineering, Applied optics, Imaging and sensing, Acoustics

## Abstract

The optical detection of nanoparticles, including viruses and bacteria, underpins many of the biological, physical and engineering sciences. However, due to their low inherent scattering, detection of these particles remains challenging, requiring complex instrumentation involving extensive sample preparation methods, especially when sensing is performed in liquid media. Here we present an easy-to-use, high-throughput, label-free and cost-effective method for detecting nanoparticles in low volumes of liquids (25 nL) on a disposable chip, using an acoustically actuated lens-free holographic system. By creating an ultrasonic standing wave in the liquid sample, placed on a low-cost glass chip, we cause deformations in a thin liquid layer (850 nm) containing the target nanoparticles (≥140 nm), resulting in the creation of localized lens-like liquid menisci. We also show that the same acoustic waves, used to create the nanolenses, can mitigate against non-specific, adventitious nanoparticle binding, without the need for complex surface chemistries acting as blocking agents.

## Abstract (web summary)

Detection of small, translucent particles is challenging due to their low inherent scattering. Here, the authors present an easy, high-throughput, label-free method for detecting nanoparticles in low volumes of liquids on a disposable chip, using an acoustically actuated lens-free holographic system.

## Introduction

The detection of nanoparticles, including the biosensing of viruses and bacteria, remains an important field in the biomedical, physical and engineering sciences, with applications as diverse as medical microbiology, cosmetics, paints, drug delivery systems and as probes for DNA sensing. Their detection, however, remains a challenge, especially during in situ liquid measurements, which often require the use of sophisticated, costly and bulky equipment coupled with advanced sample preparation methods^1,2^. Some of the most commonly used techniques for sensing such small particles are based around imaging systems including e.g., optical nanoscopy^3^, atomic force microscopy^4,5^, and resistive pulse sensing^6^. High resolution optical microscopy methods^7–9^ have also been developed for this purpose, including dynamic light scattering, nanoparticle tracking analysis^10^, as well as super-resolution^11,12^, near-field^13,14^ and dark-field^15,16^ microscopies. In many cases, these either require labeling with markers (e.g., by using fluorescent molecules, Raman active centers, or optically scattering particles), or are limited by the need for samples to be in a solid state (i.e., dry), adding additional preparation steps. As nanoparticles are not only synthesized but are also utilized in liquids^17^, their detection and imaging in solvents and aqueous media is increasingly important.

Many optical detection techniques for nanoparticles, including those listed above, are often limited in their resolution due to optical diffraction and typically only have a small field-of-view, FOV, (often <0.1 mm^2^) making measurements both time consuming and limiting, in terms of the sample volume. This in turn constrains both their sensitivity and throughput, which are key analytical parameters for many applications. Recently a microsphere-enhanced optical imaging system has been used to enable <25 nm resolution albeit with a small FOV of 125 µm × 125 μm^18^.

Lens-free sensor systems used in computational holographic imaging have overcome some of the limitations related to nanoparticle detection and characterization^19,20^, with images being computationally reconstructed from in-line holograms, resulting from the interference between the directly transmitted wave and the scattered optical wave from the sample. The resolution and detection sensitivity of the lens-free holographic sensing, however, still depend on the scattering properties of the target nanoparticles, the signal to noise ratio and the pixel size of the detector-array (where complementary metal–oxide–semiconductor, CMOS, image sensors are often chosen for their low-cost and ease-of-use)^21^. Although, the lens-free holographic imaging systems can be used for the reconstruction of 2D and 3D images, their resolution is fundamentally limited by the diffraction of light. Recent improvements in holographic imaging, including the use of pixel super-resolution techniques^22,23^ have already enabled enhanced resolution by digitally dividing individual pixels of an image sensor into much smaller virtual pixels. In other innovations, liquid polymers that self-assemble around nanoparticles have been used to create nanolenses around the nanoparticles, to mitigate against the low signal-to-noise ratio^19,24^, and have enabled the detection and sizing of sub-50 nm particles^22^.

In all these cases reported to date, where holography has been used for nanoparticle detection, the sample has been imaged and characterized in a dry state (i.e., not in a bulk liquid film), thereby constraining the throughput and range of applications. Examples of such techniques have including either draining the solvent from the chip^19^, using vapor condensation^24^, or using pyroelectric effects at high temperatures (~100 °C)^25,26^. Alternatively, where imaging has been achieved in a bulk film, this has involved using electrohydrodynamic methods to shape the fluids and create lensing structures. However, these methods are generally slow and require relatively high voltages at previously designed planar electrode arrays, producing lensing systems that generally have a small FOV^27–29^.

**Fig. 1:**
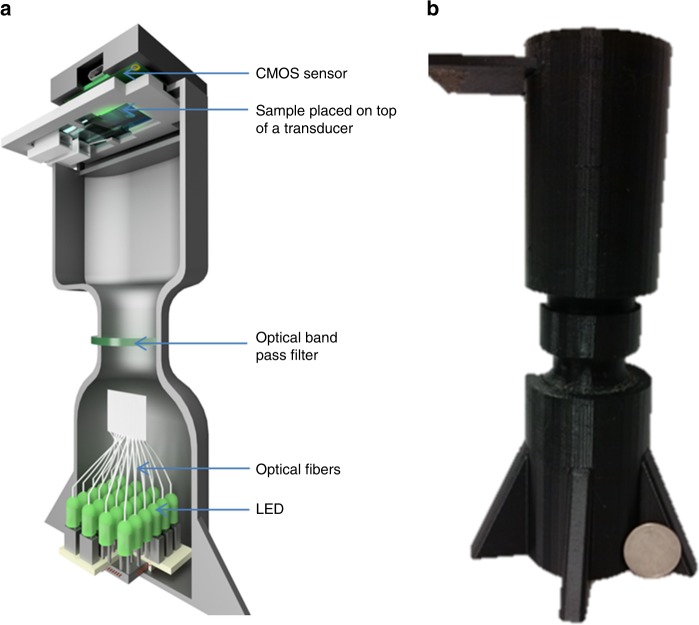
Lens-free holographic imaging system. **a** Schematic of the lens-free sensor system showing internal components of the device. The device consists of a partially coherent light-source (created by coupling the light from an LED into an optical fiber that passes through a band-pass filter) illuminating the sample before being detected by the CMOS imaging sensor. **b** The 3D-printed physical hardware, with a quarter coin placed in front of it.

**Fig. 2:**
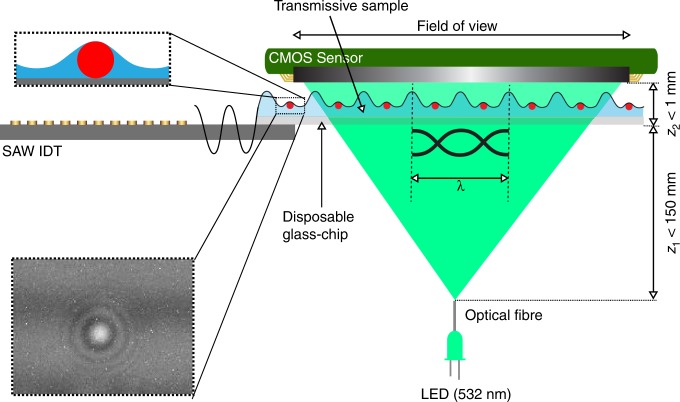
Optical system design. A standing Lamb-type wave mode transformation from a SAW transducer is illustrated. This wave spatially distorts the liquid due to pressure differences. At the antinodes, the liquid forms a thin layer over nanoparticles. This creates a localized lensing effect, enhancing the optical signal and hence the detection of nanoparticles in liquid. The height of the wave can be tuned by adjusting the input power which allows the tuning of the lensing effect.

As an alternative, here we present a low power, high-throughput, and a cost-effective method for detecting nanoparticles including bioparticles in a small liquid sample volume (~25 nL), using an acoustically actuated, lens-free holographic system, see Fig. 1. The technique enables the creation of on-demand nanolenses in liquids using carefully defined geometric and operational conditions to displace the liquid across the full FOV ~ 30 mm^2^. The acoustic pressure nodes on the glass chip form convex menisci around the nanoparticles and enhance their optical signal response, Fig. 2. We optimized the acoustic frequency and power, showing both the dispersion analysis of the guided acoustic waves in the disposable chip, as well as the power density coupling as a function of fluid height. We also analyzed the dimensions of the nanolenses as a function of nanoparticle size. By using disposable chips with immobilized antibodies, we are able to obtain the specificity required for biological sample identification. Finally, by exploiting the differential motion of the particles on the surface of the chip during acoustic actuation, which creates local streaming, we distinguish between nonspecifically bound particles (which are released from the surface upon acoustic actuation) from those which are specifically bound by antibodies (and remain fixed). The platform provides a highly adaptable, low-cost, easy-to-use, and field-portable system that has the potential to be readily integrated into different types of biosensing strategies, enabling, e.g., the high-throughput detection of viruses and bacterial cells in solutions.

## Results and discussion

### Detection system

The proposed detection method integrates both acoustical and optical functionalities onto the same platform, built around a reusable lithium niobate substrate, interfaced with a disposable glass chip. The acoustical system involves the excitation of Rayleigh waves, by an interdigitated transducer (IDT) patterned onto the reusable lithium niobate piezoelectric wafer, which interacts with the thin disposable chip (~145 µm) to generate dispersive Lamb-type guided waves which deform the liquid layer to enhance optical signatures from nanoparticles, Fig. 2. The generated guided waves in the disposable chip were analyzed analytically in the plate geometries, with or without liquid loading, enabling us to control wave propagation under different waveguiding conditions.

### Numerical analysis

**Fig. 3:**
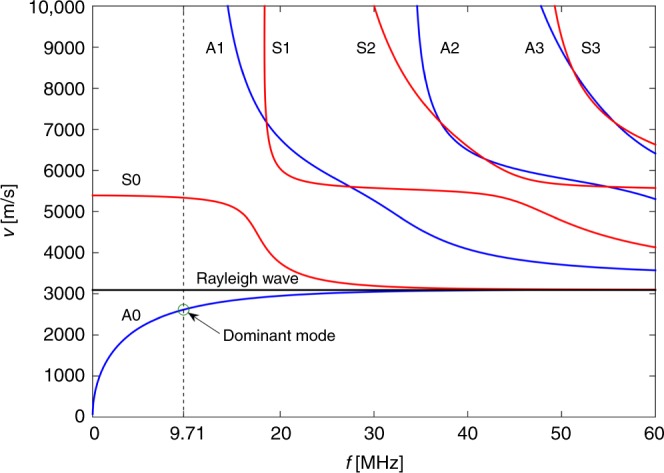
Dispersion curves for a glass plate. The variation of the phase velocity across a wide range of frequencies. We have access to two different Lamb-type waves (antisymmetric A* and symmetric S*) in a 145 µm thick glass for the frequency of operation of our device (dashed line). *A*0 is the dominant mode due to the excitation source being antisymmetric.

In detail, we consider how the guided wave propagates in a nonviscous liquid–solid bilayer with wavenumber *k* = 2*πf*/*v*, where *f* is the frequency and *v* is the guided wave phase velocity. We ignored the effect of viscosity on the phase velocity because it is negligible compared to the effect on attenuation^30^. The liquid layer has a thickness of 2*a* that can be characterized using the ultrasonic wave phase velocity in the liquid *c*_*F*_, and the volumetric mass density *ρ*_*F*_. The elastic solid layer that enables propagation of ultrasonic longitudinal and transversal waves with the velocities *c*_*L*_ and *c*_*T*_, respectively has a thickness of 2*h* and volumetric mass density *ρ*. The small thickness of the disposable glass chip (~145 µm, with respect to the wavelength) results in mode transformation of a surface acoustic wave (SAW) to a Lamb-type wave at 9.71 MHz as shown in the frequency–phase velocity plot in Fig. 3.

The propagation of guided waves in the liquid–solid bilayer was subsequently analyzed using the dispersion equation^31^

1 $$	\pi ^2\frac{{\rho _F}}{\rho }p{\mathrm{\Omega }}^4\left( {d^2\cos \left( {2p} \right)\sin \left( {2q} \right) + 4\xi ^2pq\sin \left( {2p} \right)\cos \left( {2q} \right)} \right){\mathrm{sin}}\left( {2\frac{a}{h}r} \right)\\ 	+ 16rAS\cos \left( {2\frac{a}{h}r} \right) = 0,$$

where

2 $$A = d^2\sin \left( p \right)\cos \left( q \right) + 4\xi ^2pq\cos \left( p \right)\sin \left( q \right),$$

3 $$S = d^2\cos \left( p \right)\sin \left( q \right) + 4\xi ^2pq\sin \left( p \right)\cos \left( q \right).$$

Eqs. (2) and (3) represent the dispersion relations for the antisymmetric and symmetric guided modes (Lamb-type wave) in the free solid plate. The remaining coefficients are given by

$$p = 2{\uppi}fh\sqrt {\frac{1}{{c_L^2}} - \frac{1}{{v^2}}} ,$$

$$q = 2{\uppi}fh\sqrt {\frac{1}{{c_T^2}} - \frac{1}{{v^2}}} ,$$

$$r = 2{\uppi}fh\sqrt {\frac{1}{{c_F^2}} - \frac{1}{{v^2}}} ,$$

$$d = 8{\uppi}f^2h^2\left( {\frac{1}{{c_T^2}} - \frac{2}{{v^2}}} \right),$$

where $${\mathrm{\Omega }} = \frac{{4fh}}{{{\mathbf{c}}_{\mathbf{T}}}},\xi = \frac{{4fh}}{{\mathbf{v}}}.$$

An impedance mismatch causes the Lamb-type wave to reflect back from the glass–air boundary, hence creating a standing wave. Numerical results show that for a ~1 µm thick layer of liquid (with properties detailed in Supplementary Table 1) on top of the glass chip, the thin plate can support only zeroth-order (fundamental) Lamb-type wave modes *A*0 and *S*0 at the excitation frequency of 9.71 MHz (the cut-off frequency of the higher order mode is 11.70 MHz). As the excitation frequency was increased, higher-order modes could be excited and the fundamental modes converged to a surface wave at a high frequency limit of 50 MHz, Fig. 3.

**Fig. 4:**
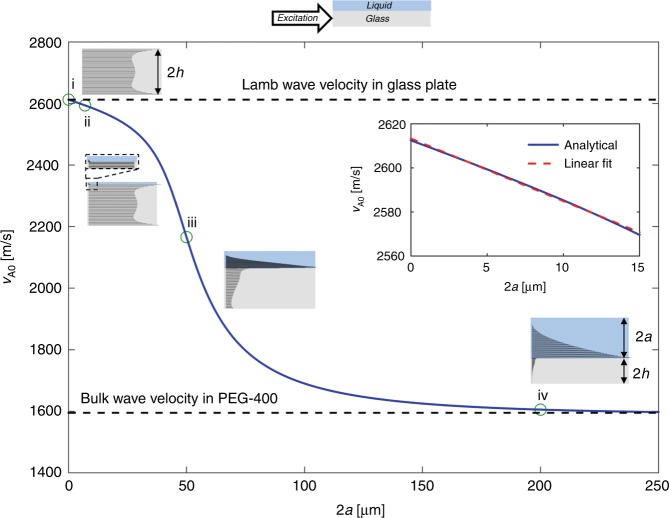
Wave properties at the interface of a glass plate with different thicknesses of liquid loading. The phase velocity and the corresponding power density plot of liquid loading on a glass plate at different thickness (i) 0 µm, (ii) 1 µm, (iii) 50 µm, and (iv) 200 µm. The inset shows that when the liquid layer thickness is small (2*a* ≪ 2*h*), the Lamb-type wave coupling into the liquid is small. When the layer thickness is such that (2*a* < 15 μm), the guided wave wavelength of the antisymmetric mode decreases linearly from *λ* = 269.1 μm to *λ* = 264.6 μm and can be represented by a linear expression *λ*_A0_ = −5.674*a* + 2613.3 μm (*R*^2^ = 0.99).

Similarly, as thickness of the liquid loading was increased, the Lamb wave started to couple into the liquid and more energy leaked into the sample, as shown in the power density flow curves, Fig. 4. The direction of power flow indicates the wave propagation direction across the thickness of the material, and the length indicates the magnitude. For a thin liquid film, e.g., ~1 µm, when 2*a* < 2*h*, a small leakage of the wave energy in the liquid was possible. In contrast, as the thickness was increased, e.g., to 200 µm, such that 2*a* > 2*h*, the extent of wave energy leakage into the liquid increased, resulting in acoustic streaming in the liquid, Fig. 4. Thus, by ensuring that the sample thickness satisfied the condition 2*a* ≪ 2*h*, we could also ensure that the Lamb wave has minimal leakage into the liquid, limiting the distortions of the liquid layer on top of the glass to those induced mechanically by the deformations of the plate (the liquid–solid bilayer), rather than those caused by convection, during acoustic streaming.

Nanoparticle detection with our in-line holographic system will depend in part upon the nature of the nanoparticle-nanolens complex and its scattering properties, as well as on the pixel size and the signal to noise ratio of the image sensor-array used. The light scattering intensity from nanoparticles that are significantly smaller than the wavelength of light used, depends on the sixth power of the size of the particle, and is governed by Rayleigh scattering accordingly^32^

4 $$I = I_o\frac{{1 + {\mathrm{cos}}^2\theta }}{{2r^2}}\left( {\frac{{2{\uppi}}}{{\uplambda }}} \right)^4\left( {\frac{{n^2 - 1}}{{n^2 + 2}}} \right)^2R_p^6,$$

where *I*_*o*_ and *λ* are the incident intensity and the wavelength of the light source, respectively, *r* is the distance of the observer, *R*_*p*_ is the radius of the particle, *n* is the refractive index of the particle relative to the background, and *θ* is the scattering angle.

Thus, nanoparticles are difficult to detect due to their low optical scattering cross-section. In common with other coherent interferometric systems, for holographic sensors, the field signal scales as a third power of the size, thus providing a stronger signal for nanoparticle detection^33^. The radial coordinate of the meniscus of the nanolens (*r*_*m*_), as a function of the elevation above the substrate was determined by solving the Young-Laplace equation^19^

5 $$r_m\left( z \right) = \frac{1}{{\zeta d}}{\mathrm{cosh}}[\zeta \left( {dz + 1} \right)],$$

where

$$\zeta =	 \, -\!{\mathrm{arcsinh}}\left( {{\mathrm{cot}}\,\theta _s} \right),\\ d = 	\, \frac{1}{{z_0}}\left[ {\frac{1}{\zeta }{\mathrm{arcsinh}}\left( {\frac{{\beta \left( {z_o} \right){\mathrm{cos}}\,\theta _p - {\mathrm{sin}}\,\theta _p}}{{{\mathrm{cos}}\,\theta _p + \beta \left( {z_o} \right){\mathrm{sin}}\,\theta _p}}} \right) - 1} \right],\\ \beta \left( {z_o} \right) = 	\, \frac{{R_p - z_o}}{{\sqrt {R_p^2 - (R_p - z_o)^2} }},$$

and *θ*_*s*_ and *θ*_*p*_ are the contact angles of the substrate and a spherical particle respectively, *z*_0_ is the elevation of the meniscus–particle contact line.

These equations show that the size of the nanolens meniscus depends upon the contact angle which in turn depends upon the properties of the liquid, the particles and the chemical nature of the surface of the chip. Given the very small length scales involved, it is difficult to measure the contact angles in our system^34^. Although, the nanoparticle itself is small, the nanoparticle–nanolens complex itself is relatively large. We therefore use approximations from previously reported simulations of nanolenses^19^ of similar length scales.

### Particle detection

Based upon this understanding, model experiments were performed using spherical commercially sourced polystyrene nanoparticles (with diameters between 140 nm and 1 μm) with well-controlled physical and chemical properties such as the refractive index, density, and shape. The liquid sample was back-illuminated using a partially coherent light source and the in-line holograms, resulting from the interference between directly transmitted wave and the scattered object wave, were recorded using a low-cost CMOS image sensor. The sample was placed close to the image sensor at a distance of ~1 mm.

**Fig. 5:**
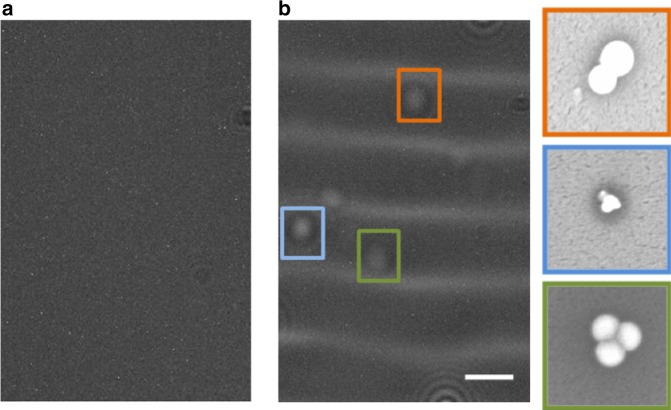
Nanoparticle detection. Lens-free holographic detection of 200 nm polystyrene nanoparticles both **a** before and **b** after acoustic actuation. Scale bar for the lensfree image is 100 µm. The colored insets in (**b**) show the SEM images of the corresponding nanoparticles. The field-of-view for SEM images is 1 µm × 1 µm.

Figure 5 shows the detection of 200 nm biotinylated polystyrene nanoparticles bound onto streptavidin, itself immobilized onto the glass surface^20^. No nanoparticles could be detected prior to the acoustic actuation, Fig. 5a. Upon generation of the SAW, the nanoparticle–nanolens complexes were formed at the nodes of the waves, Fig. 2, and nanoparticles were detected, Fig. 5b. Figure 5b inset also shows the SEM images (as a gold standard reference imaging method) of the 200 nm particles. When the SAW excitation was switched off, the Lamb wave on the disposable chip dissipated immediately and the resulting relaxation of the liquid film resulted in the particles becoming invisible.

**Fig. 6:**
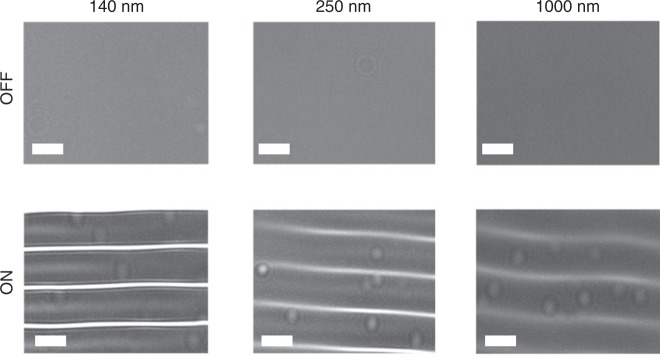
Nanoparticle detection experiments with and without SAW. Images of 140–1000 nm nanoparticles are reported. Without SAW there is no standing wave and the nanoparticles cannot be detected by the CMOS image sensor. Application of SAW creates a thin film meniscus around the nanoparticles and so their lens-free holograms can be detected. The scale bar in all images is 100 μm.

Similarly, Fig. 6 shows the range of nanoparticle sizes that we were able to detect between 140 nm and 1 μm, with acoustic actuation of the optofluidic lensing switched on and off. We were unable to detect nanoparticles that were smaller than 140 nm, establishing our limit of detection for the holographic sensor system at an illumination wavelength of 532 nm. In principle, tuning of the illumination wavelength makes it possible to increase the scattering cross-section of nanoparticles, although in our case, the signal to noise ratio improvement remained limited.

**Fig. 7:**
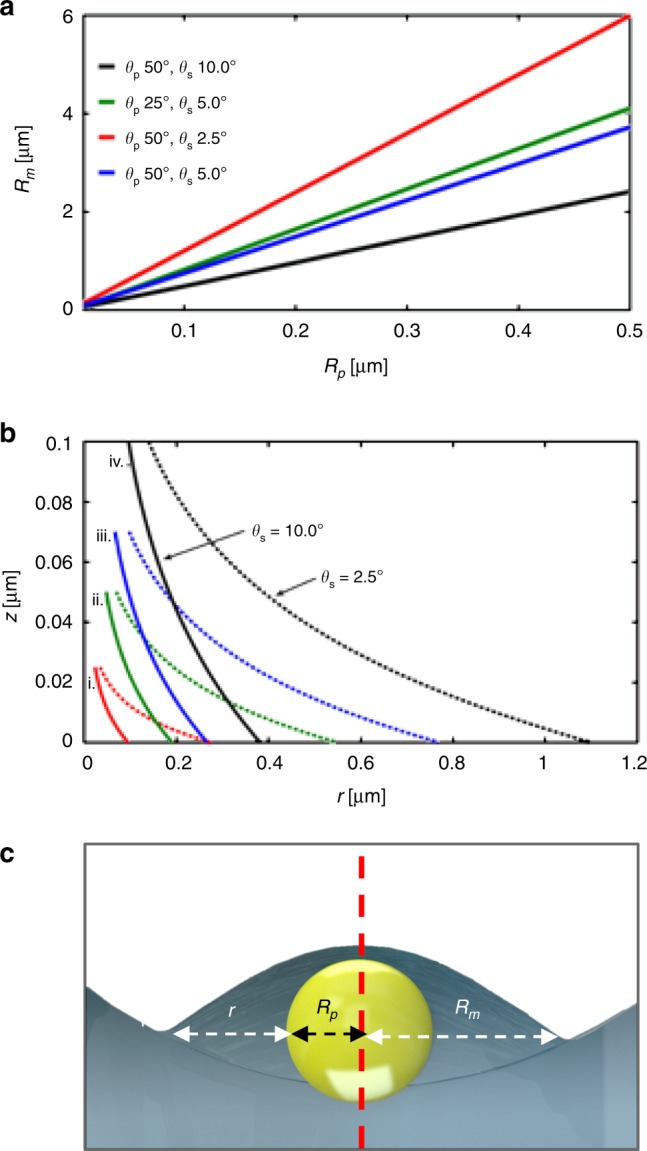
Parametric analysis of nano-lens curvature for different particle sizes. **a** Numerical calculations of nanoparticle-nanolens complex radius, *R*_*m*_ as a function of particle size *R*_*p*_. Each line represents calculations for different contact angle pairs *θ*_*s*_ and *θ*_*p*_. For a 200 nm nanoparticle, the corresponding maximum *R*_*m*_ can exceed 2 μm. **b** Numerically calculated meniscus radius for (i) 50 nm (red), (ii) 100 nm (green), (iii) 140 nm (blue), and (iv) 200 nm (black) spherical nanoparticles to emulate the nanoparticles used experimentally (also see Supplementary Fig. 1). The unbroken line (-) represents *θ*_*s*_ of 10.0° whereas, the broken line (--) represents *θ*_*s*_ of 2.5°. For a 200 nm nanoparticle, the corresponding maximum *R*_*m*_ can exceed 2 μm. **c** Schematic diagram of the nanolens parameters. The red dotted indicates symmetry.

Our numerical calculations (Fig. 7a) show that for a 140 nm diameter spherical particle, the radius of the nanoparticle–nanolens complex *R*_*m*_ can range between 0.345 and 0.845 µm. To understand the capability of the detection system to resolve closely spaced objects for a transmission based optical holographic systems, however, we have to consider both the size of nanoparticle–nanolens complex (*R*_*m*_) and the pixel size of the CMOS sensor, which is 1.67 µm in our case. Although it has previously proved possible to circumvent any limit imposed by the sensor’s pixel size (e.g., by using pixel super-resolution techniques)^27^, in this detection system we use only simple in-line holographic detection. Thus, if the inter-particle spacing is smaller than the size of the individual nanolens complex, then proximal particles are likely to merge into a single detection point. At our detection limit, observing 140 nm diameter particles, the total particle–lens complex diameter ranges from 0.690 to 1.690 µm (depending upon the contact angles), which is on the order of the pixel size of the CMOS imager in our setup.

A further key analytical parameter in the analysis of our detection system is an understanding of its throughput. For conventional far-field microscopy using a high magnification objective, particles of comparable size (~200 nm) can be readily detected over an FOV of 0.5–1 mm^2^. Our holographic detection system, however, has an image sensor area of 30 mm^2^, offering a potential improvement in throughput by a factor of ~50-fold. Further, and unlike conventional far-field optical detection, we can improve our FOV and hence our throughput by using even larger sensors (without any reduction in our ability to detect the particles).

**Fig. 8:**
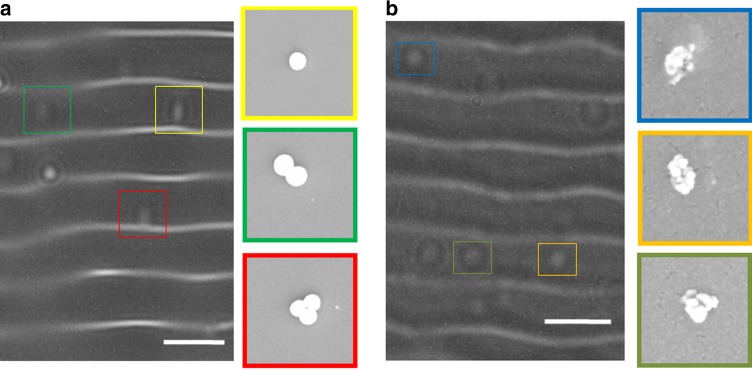
Bacteria and virus detection. Acoustically actuated lens-free detection of **a** *Staphylococcus aureus* bacterial cells and **b** herpes simplex virus particles on a glass substrate, immobilized using specific antibodies. The scale bar for lens-free images is 100 µm. The insets (colored squares) show the SEM images of the corresponding bioparticles. The SEM field-of-view is 5 µm × 5 µm for bacterial cells (**a**) and 1 µm × 1 µm for virus particles (**b**).

The combination of size of the nano-lenses and the pixel resolution also governs the optimal density per unit area of the disposable chip, a process that can be controlled by the nature and density of the ligand-binding moieties, which in this study included both antibodies and streptavidin (together with cross linkers to ensure that an optimal distance between the particles is maintained). Following optimization of these parameters, the sensor was able to detect bioparticles in thin liquid films, including herpes simplex virus (HSV-I) (~200 nm)^20^ and *Staphylococcus aureus* (*S. aureus*) bacteria (800–1000 nm)^35,36^, Fig. 8a, b, respectively. Detecting such bioparticles in liquids is a challenging task due to the small optical scattering cross-section and refractive index difference with the surrounding liquid^19^. In both cases the bioparticles were captured on the disposable chip, using appropriate antibodies immobilized with silane chemistries, see Experimental Section. SEM images of the pathogens are given in the insets of Fig. 8.

The detection of these pathogens was chosen due to their importance in the field of microbiology and infectious disease. HSV is one of the most widespread sexually transmitted diseases around the globe, which has been estimated to have affected more than 50% of the adult population in the US alone^37^. Similarly, *S. aureus* is a highly infectious pathogen that causes skin infections, respiratory infections, and food poisoning.

### Removal of nonspecific binding

When relying upon any ligand binding strategy within an analytical systems to improve the specificity of detection (e.g., in our case by exploiting antibody–antigen binding or streptavidin–biotin avidity) nonspecific binding events, including, the adventitious binding of beads, viruses or nanoparticles on the chip surface, can lead to false positives. Previously established methods to minimize nonspecific binding can include coating of chips with albumin, polyethylene glycol (PEG), or other blocking polymers and macromolecules^38^, all of which adds to the complexity of chip manufacture.

**Fig. 9:**
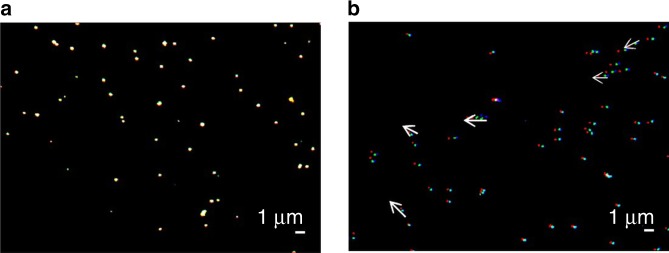
Removal of non-specific binding using SAW. A comparison between the movement of **a** specifically bound and **b** nonspecifically bound particles (biotinylated fluorescent nanoparticles), following acoustic actuation (9.71 MHz, 220 mVpp input amplitude). The images were captured by fluorescence microscopy. The false color coding is as follows: particles at *t* = 0 s are denoted in blue, at *t* = 2 s in green, and at *t* = 8 s in red. The arrows indicate the direction of the movement.

It is already known that nonspecifically bound particles^39^ and proteins^40^ can be removed using acoustic actuation with surface waves. Building upon this knowledge, we chose to integrate the acoustic actuation of the nanolens with a strategy to mitigate against nonspecific binding. Figure 9a shows superimposed fluorescent images of specifically bound fluorescently labeled 1000 nm polystyrene nanoparticles (which do not move under acoustic actuation) whilst nonspecifically bound particles showed significant movement.

The mechanism of this controlled movement will depend upon the size of the particle. For smaller particles (<500 nm), actuation will arise due to acoustic radiation pressure, whilst for larger particles (>500 nm) movement is most likely to be due to acoustically induced flows near the liquid–solid boundary layer^41^, Fig. 9b. We developed a simple algorithm based upon the superimposition of three images, that enabled us to readily identify specifically bound and nonspecifically bound particles, by establishing an absolute threshold for the distance moved (a parameter that we determined experimentally based upon the approximate size of the target particles, liquid’s viscosity, and acoustic power).

Our overall ability to detect particles is fundamentally determined by the effective signal to noise ratio of the imaging system, the properties of the nanoparticle and nanolens, including e.g., the size of the particle and the lens complex, the pixel size, the refractive properties of the nanoparticle and surrounding medium (as the optical contrast), the height of the liquid film, and the nature of the acoustic waves. The system itself is complex, and also includes a series of related factors, e.g., surface properties of the particle and the chip, nature of the supporting media, the residual liquid height at the nodes and the antinodes, the local temperature, the power of the SAW at that particular location and the position of the nanoparticles relative to the node and antinode.

In the present configuration, in Fig. 6, we see that nanoparticles are detected both at pressure nodes and at the antinodes, and we believe that only a very low number of false-negatives exist. We note that, by varying the excitation frequency, ultrasonic standing waves of different wavelengths can be created which spatially shift the position of nodes and antinodes in the liquid. Capturing images of the sample at different acoustic frequencies and superimposing them would uncover any blind spots. An alternative approach, to improve the coverage and mitigate against any blind spots, would be to configure two IDTs placed orthogonal to each other, to create two dimensional nanolens array thereby increasing the sensing area and throughput of the system.

We also note that the technique is sensitive both to the shape and size of the target nanoparticles. The technique is therefore able to differentiate between clusters of particles and the same particles as individual objects, but it is not able to differentiate between small particles aggregated in clusters as this will be detected as a single (larger) particle, when using the raw hologram for rapid detection. In any mixed sample of different nanoparticles, particularly when they are biological, we propose that the strategy for detection be best performed with an immobilized ligand, such as an antibody as demonstrated in Figs. 8 and 9.

In conclusion, we presented an acousto-optic sensing method that enables the detection of nanoparticles in liquid samples using a low-cost, mass producible, wide FOV, high-throughput, and portable lens-free optical microscope coupled with ultrasonic actuation on a disposable chip. We demonstrated the creation of on-demand dynamic nanolenses using an active substrate without moving parts and thus favouring the miniaturization of the optical systems. We showed that a thin meniscus forms around the nanoparticles, which acts as a nanolens, by generating standing waves in the liquid. As a practical application, the detection of the HSV-I virus (200 nm diameter) as well as *S. aureus* bacterial cells (800–1000 nm diameter) in liquid was also demonstrated.

In principle, the technique described requires only simple sample preparation. However, to enable detection outside of the laboratory environment, as used in this study, where the sample composition (including the opaque nature of many matrices), the stage in the life cycle of microbes, and-or the state of bioparticle aggregation (due to matrix effects), may all represent challenges that could affect the analytical metrics, particularly around sensitivity, more detailed optimization will be necessary. In order to mitigate against these, we demonstrated that the technique can be combined with controlled antibody immobilization methods to enable specifically bound particles to be detected, whilst nonspecifically bound particles can be removed through acoustic streaming^39,40^.

## Methods

### Materials

Biotin and m-PEG-silane (PJK-1915 and PLS-2013) was purchased from Creative PEGWorks (NC). The fluorescently labeled nanoparticles (F-8767, F8768, and F13081), the biotin tagged HSV type 1/2 polyclonal antibody (PA1-7488), biotin tagged *S. aureus* polyclonal antibody (PA1-73174), PEG-300 and PEG-400 were acquired from Thermo Fischer Scientific. The viral culture (NATtrol Herpes Simplex Virus Type 1 Strain: MacIntyre, 50,000 copies/ml) was purchased from ZeptoMetrix Corporation. The *S. aureus* samples were bought from ATCC (ATCC 27660). The washing buffer comprised 50 mM phosphate buffer, pH 7.4. Unless otherwise stated, reagents were purchased from Sigma-Aldrich. All the materials were used as received.

### SAW device fabrication and characterization

Single crystal lithium niobate piezoelectric wafers were used to generate SAW but were not required for their propagation. In this case, the Rayleigh wave, excited on a 128° Y-cut X-propagating lithium niobate wafer with thickness of 1 mm (Roditi, UK), was coupled into a disposable glass chip (a thin microscope coverslip) as a Lamb wave. This excitation of SAW was achieved by means of an IDT with a resonant frequency of 9.71 MHz (the resonant frequency is dependent on the width of the IDT electrode, was one-fourth of device pitch, calculated using a simple frequency–wavelength relationship, *f* = c/*λ*, where, *f* is the frequency, and *c* is the sound wave propagation velocity in lithium niobate). For a 9.71 MHz IDT, *D* was 411 µm. The IDT aperture was 20 mm, and 40 titanium–gold finger pairs were fabricated on the wafer. The IDT was manufactured by standard photolithographic techniques, as has been reported previously^42^. To characterize the frequency response, i.e., the resonant frequency and the harmonics, a network analyzer (E507C ENA series, Agilent Technologies) was used.

### Lens-free detection

A schematic of the lens-free holographic sensor is shown in Fig. 1. The system was constructed from 3D-printed parts using a Dimension Elite 3D printer (Stratasys). The illumination chamber consisted of 24 fiber-coupled LEDs, centered around 532 nm, controlled by a microcontroller. The light from the fibers passed through a band pass filter with a center wavelength of 532 nm (10 nm bandwidth) before illuminating the sample. The sample was placed at a distance of ~15 cm from the light source and ~1 mm from the CMOS chip (10-megapixel CMOS image sensor (UI-1492LE-M, Imaging Development Systems) with USB readout, with an active sensing area of ~5 mm × 6 mm). It was noted that for detection of particles with PEG-based samples, the optical contrast between the polystyrene bead (*n* = 1.59) and the surrounding medium (*n* = 1.47), was small, underlining the analytical challenge.

The glass chip was placed on a lithium niobate piezoelectric wafer which had a photolithographically patterned IDT generating a 9.71 MHz acoustic wave, as described above. A 20 μL drop of PEG-400 was used to couple the ultrasonic signal between the piezoelectric wafer and the glass chip. The IDT was connected to a 50 MHz function generator (TG5011, TTi) in conjunction with an amplifier (ZHL-5W-1, Mini Circuits) and a 3A, ±24 V dc power supply. The particle detection systems and the illumination module were controlled with a software application described elsewhere^27^.

### Sample preparation on disposable chips

The chips were prepared according to previously established protocols^20^. A standard microscopy glass coverslip served as the low-cost disposable chip, which was first washed in acetone and then thoroughly rinsed, with isopropanol and deionized water. The chips were then plasma-treated for 5 min to make their surface hydrophilic, and able to wet when either reagents for immobilization or the liquid sample were added.

In those experiments where a surface functionalization was performed, this involved first adding a silane–PEG–biotin solution [1 mg/ml in a solution containing 95% ethanol (1.875 ml) and acetic acid (125 µl)] to the disposable chip (glass). After 1 hour of incubation, the surface was again washed with ethanol and water to remove unbound PEG. We then added a solution of streptavidin (100 µg/ml in water) and again incubated it for 1 hour to allow it to bind effectively to biotin–PEG. Unattached streptavidin was washed away with washing buffer. The substrate was either used immediately or stored at −20 °C for future use.

In a series of experiments exploring potential applications, we chose fluorescent biotinylated native (unmodified) nanoparticles (with diameters of 140–1000 nm, (F-8767, F8768, and F13081)), which we used as model systems to characterize the detection process. HSV-I virus particles bound to a biotin-tagged antibody (PA1-7488)) or *S.* *aureus* bacteria bound to a biotin-tagged antibody (PA1-73174) were also used to demonstrate the detection of important microbes. In both cases particles were incubated on the substrate for an hour. The samples were subsequently washed gently to remove unattached particles and then covered with a droplet of washing buffer.

The antibodies were bound to the virus particles or bacterial cells by mixing the biotin-tagged antibody (volume ratio 1:10) with the respective pathogen and by incubating the mixture for 2 hours; the unbound antibodies were washed away by centrifugal filtration. Here, we used 50 µL solution of bacteria/virus on each glass coverslip.

Immediately prior to imaging, 50 µL of PEG-400 was added onto the coverslip and spin-coated at 12,000 rpm enabling formation of a thin liquid film. The height of the liquid film (2a), was characterized using ellipsometry. Chips (18 × 18 mm) were plasma treated in an oxygen asher (PlasmaFab 505, Electroetch) and coated with PEG-400 at a range of spin-speeds, using a spinner (PWM32, Headway Research). An ellipsometer (M-2000, J.A. Woollam) was used to scan the sample at an incidence angle of 75° with polarized light. The data were analyzed using CompleteEASE software with a Cauchy model.

Acoustic actuation of particles and their subsequent movement involved fluorescence imaging (×20 Objective size, NA = 0.4, with an Olympus, BX51 microscope). Both videos and images were acquired using a charge-coupled device camera (QIMAGING, Retiga 2000R) with a 20× objective lens (*λ*_ex_ = 488 nm, *λ*_em_ = 532 nm). The images were analyzed in ImageJ^43^ (v1.5) using built-in image calculator function where the time-series images were superimposed onto each other. This simple method enabled us to readily identify specifically bound and nonspecifically bound particles, by establishing thresholds for the distance moved (a parameter determined experimentally based on the approximate size of the particles, liquid’s viscosity and acoustic power).

### Electron microscopy

Electron microscopy was used for validation and calibration purposes. The samples were imaged using an SEM (Nova 600 SEM/FIB System). Prior to holographic detection, we placed identifiable calibration marks on the glass chip using marker pens, which are easily recognizable in the lens-free images as well as in SEM images. Easily identifiable (large) objects, located close to the region of interest, were also used as secondary reference points. The horizontal and vertical distances of the particles of interest from such features were calculated using the lens-free image, to generate a map^20^. While imaging, the samples were aligned such that the angular orientation of each slide matched the lens-free image. The particles of interest were identified by locating the primary and secondary markers using the generated map. This step-by-step particle searching strategy enabled us to register our images accurately, which was used for comparison purposes.

### Numerical analysis

The dispersion curves were numerically calculated in MATLAB. The plate geometry was chosen such that *x*_3_ < 0 corresponded to a glass plate of thickness 2 *h*, and *x*_3_ > 0 corresponded to a liquid layer of thickness 2*a*. For layer *x*_3_ > 0, the liquids have a thickness (2*a*).

## Supplementary information

Supplementary Information

## Data Availability

The data including numerical simulation codes of this study are available at 10.5525/gla.researchdata.908.
