# Supplementary material for: Holographic detection of nanoparticles using acoustically actuated nanolenses
Source: Nat Commun. 2020 Jan 16;11:171. doi: 10.1038/s41467-019-13802-1 (PMC6965092; doi:10.1038/s41467-019-13802-1)
Supplement: Supplementary file 1 — Supplementary Information [file 41467_2019_13802_MOESM1_ESM.pdf]

Holographic Detection of Nanoparticles using Acoustically Actuated Nanolenses

Ray and Khalid et al.

Supplementary Information

Supplementary Information contains one Supplementary Figure and one Supplementary Table.

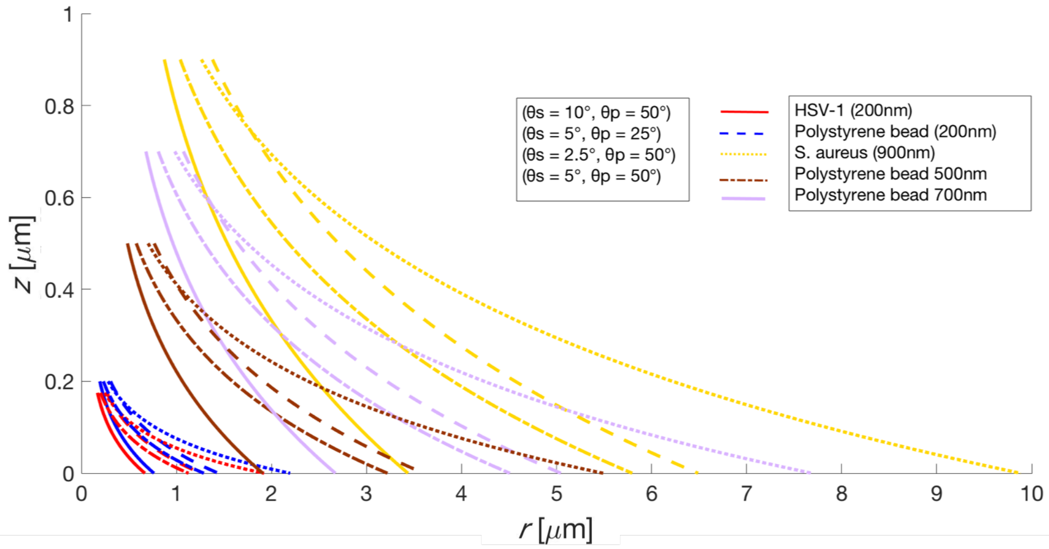

Supplementary Figure 1. Minimal surface catenoid lens shapes of the liquid nanolens formed around spherical nanoparticles. Increasing particle size results in larger nanolens radius. Each color represents a particle of different size comparable to the particles used in this paper. The different line patterns represent different contact angle pairs θ_s and θ_p . Increasing particle size results in larger catenoid lens radius. The shape of the lens is affected by θ_s and θ_p. Smaller θ_s results in larger catenoid lens radius. The calculations can be used to estimate the resolution of the system i.e. the minimum distance between the two nanoparticle-nanolens complexes required to individually detect a single nanoparticle.

Supplementary Table 1. Parameters of the spin coating of the liquid layer.

Liquid	Spin speed [rpm]	Ramp-up time [s]	Acceleration [ms^{-2}]	Dwell Time [s]	Ramp-down time [s]	Time [s]	Height [μm]
Glycerol/ PEG-400	8000 - 12000	10	500	40	10	60	~ 0.8 - 7
